# High DND1 Level Indicates a Poor Prognostic Factor in Prostate Cancer

**DOI:** 10.1155/2021/9948241

**Published:** 2021-10-22

**Authors:** Gongquan Xu, Xiaotong Yang, Chunchang Li, Fan Wang, Jing Cui, Bin Li, Huiyuan Xiao, Kunlong Tang, Zhe Cui

**Affiliations:** Department of Urology, Tianjin Medical University General Hospital, Tianjin, China

## Abstract

**Background:**

Dead end 1 (DND1) plays a vital role during oncogenesis and cancer progression by regulating the mRNA content via competitive combination with miRNA, but what function it exerts in prostate cancer has been unclear. The purpose of this paper is to explore the correlation between DND1 expression levels and clinical characteristics in prostate cancer (PCa) patients.

**Materials and Methods:**

To assess the expression of DND1 in tumor specimens compared with paired paracancerous tissues, the sample from 83 patients was analyzed by immunohistochemistry. The Cancer Genome Atlas (TCGA) database was used to verify our results. Subsequently, we statistically analyzed the relationship between DND1 expression and the clinical prognosis of PCa patients.

**Results:**

Compared with paracancerous tissues, DND1 has a higher expression level in prostate cancer. The overexpression of DND1 in protein level was significantly associated with the higher clinical stage (*P* = 0.006), ISUP grading group (*P* < 0.001), seminal vesicle invasion (*P* = 0.006), and PSA density (*P* = 0.002). Furthermore, the overexpression of DND1 indicates a poor clinical prognosis in prostate cancer patients.

**Conclusion:**

High-level expression of DND1 was associated with tumor progression and poor clinical prognosis. Hence, DND1 may become a potential prognostic biomarker for PCa.

## 1. Introduction

Prostate cancer (PCa) is one of the most prevalent solid malignant tumors that threaten men's health, and the morbidity of prostate cancer ranks the top two among all-male tumors in European countries (1, 2). In the United States, prostate cancer is a significant public health burden and it is estimated that an additional 180890 cases were diagnosed in 2016 (3). With more Westernized diets and lifestyles, the incidence of prostate cancer in Asian countries has also continued to rise in the last 10 years (4). While some clinical examinations such as the prostate-specific antigen (PSA) value, imaging diagnosis, and Gleason score can assess the patient's condition to a certain extent, they cannot clearly indicate the prognosis of each patient (5). The improper treatment based on these clinical operations greatly harms the patient.

In the last decade, due to PSA screening and prostate biopsy, an increasing number of prostate cancer patients have been diagnosed (6). Given the heterogeneity of prostate cancer (7), it is necessary to seek potential prognostic factors to guide the pretreatment decision-making process for increasing the prostate cancer subgroup (8). Besides, novel biomarkers might be of great clinical value if they could help in offering a patient the beneficial individual treatment strategy for advanced tumor stages (9). Facing clinical challenges in the urology field, the need for emerging reliable biomarkers in prostate cancer treatment is urgent.

DND1, an evolutionary conserved RNA-binding protein (RBP) (10, 11), is necessary for the differentiation of primordial germ cells (PGCs) and the suppression of germ cell tumors and plays a vital role in regulating male germ cell development (12) through the interplay with Nanos2 and Nanos3 (13, 14). A piece of increasing evidence presents that DND1 acts as a wider function in human cancers such as primary acute myeloid leukemia (AML), colorectal cancer (CRC), and hepatocellular carcinoma. Data from one study suggested that the expression of RNA-binding proteins RBM38 and DND1 was suppressed in primary AML patients (15). The downregulation of DND1 expression caused by ectopic expression of miR-24 in SW48 cells, one of CSC cells, results in inhibition of SW48 cell proliferation (16). Furthermore, DND1 acts as a tumor suppressor by restraining CSC-like characteristics by activating the Hippo pathway in HCC cells (17). Indeed, DND1 plays an essential role during oncogenesis and cancer progression. However, no institution or individual has studied the expression level on the protein level and biological effects of DND1 in prostate cancer.

In this study, we found that there was a difference in the mRNA level of DND1 between PCa tissues and adjacent prostate tissues in the cancer genome map (TCGA) dataset by bioinformatics analysis. Then, we used the immunohistochemical method to verify the expression level of DND1 protein in PCa and adjacent prostate tissues and studied the relationship between the increased expression of DND1 and the clinical characteristics of PCa patients. Besides, we used bioinformatics to evaluate the effect of DND1 expression on disease-free and overall survival in patients with PCa. The results of this research show that DND1 is of great significance in the diagnosis and prognosis of patients with prostate cancer.

## 2. Materials and Methods

### 2.1. Patients and Tissue Samples

This study was approved by the Ethics Committee of Tianjin Medical University General Hospital, China. The clinical research informed consent was signed by each patient before surgery. A total of 83 prostate cancer samples were collected in the process of prostate targeted biopsy from June 2018 to March 2021. All prostate cancer patients received radical prostatectomy in Tianjin Medical University General Hospital, Tianjin, China, after the pathological results returned, and were classified according to the 2017 Union for International Cancer Control (UICC) TNM staging. All tissue samples were preserved in formalin solution for immunohistochemical analysis. Patients with a history of preoperative androgen deprivation, chemotherapy, or radiotherapy did not meet the inclusion criteria. The clinic parameters including age, preoperative prostate-specific antigen (PSA), PSA density, clinical stage, Gleason score, lymph node metastasis, seminal vesicle invasion, and bone metastasis were collected completely.

### 2.2. Bioinformatics Analysis

The GEPIA website (http://gepia.cancer-pku.cn/index.html) is used to analyze data on DND1 from TCGA public database. Data for differential genetic analysis from TCGA public database (including 498 PCa tissues and 52 noncancerous prostate tissues) were accessed. The disease-free survival and overall survival time were obtained from TCGA public database and were collected to analyze the association between the expression of DND1 at the mRNA level and patients' disease-free survival and overall survival.

### 2.3. Immunohistochemistry and Scoring

To observe the expression of DND1 at the protein level, we performed standard immunoperoxidase staining procedures. Briefly, 5 *μ*m sections were obtained from the FFPE sample. First, the paraffin sections were dewaxed in xylene and then rehydrated in 100%, 95%, 80%, and 60% ethanol. Then, immerse the slices in the citric acid repair solution and place the slices in the microwave oven for 2 repairs at medium heat for 5 minutes each time. The slides were then incubated with 3% hydrogen peroxide for 10 minutes at 37°C to inactivate endogenous peroxidase. The sections were blocked with 5% blocking serum for 1 hour at room temperature. The tissue was incubated with the primary rabbit/mouse anti-human monoclonal antibody DND1 antibody at 4°C overnight. After washing with TBS, the tissues were incubated with anti-rabbit/mouse HRP-labeled polymer as the secondary antibody at 37°C for 30 minutes and rinsed with TBS. Then, the sections were dripped with 3,3′-diaminobenzidine (DAB) until the sections were developed. After that, tissues were redyed in hematoxylin for 3 minutes and dehydrated with alcohol (60-100%) for each grade for 5 minutes. After taking it out, put it in xylene for 10 minutes, twice. The slide was mounted with mounting glue and observed with a microscope. Negative control sections were stained as described but with the primary antibody omitted.

The expression content of DND1 at the protein level is semiquantitatively estimated by the sum of the scores of the proportion of positive staining cells and staining intensity. The scores for the proportion of positively stained cells are as follows: 0, <5% positive tumor cells; 1, 5%~50% positive tumor cells; and 2, >50% positive tumor cells. The staining intensity scores are as follows: 0, negative staining or weak staining; 1, medium staining; and 2, intense staining. The sum of 2 parameters indicates the expression level: 0~2: low level and 3~4: high level. The scores were estimated independently by two experienced pathologists, and the average of the scores was considered as the final assessment score. The controversial result was reassessed by another senior pathologist.

### 2.4. Statistical Analysis

All statistical analysis uses SPSS 23.0 statistical software (SPSS Inc.). The variables of clinicopathological parameters and the scores of DND1 expression at the protein level were assessed by using the chi-square test. Kaplan-Meier curve (GEPIA website) was used for survival analysis. *P* value descriptive data were generated for variables. *P* < 0.05 was considered statistically significant.

## 3. Results

### 3.1. DND1 mRNA Level in PCa Tissues and Normal Tissues

**Figure 1:**
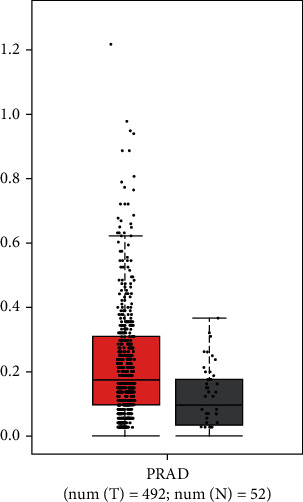
Based on bioinformatics analysis from TCGA database, the expression of DND1 mRNA level in prostate cancer tissues was higher than that of normal prostate tissues.

To validate the accuracy of our data, TCGA database was used to analyze the expression of DND1 mRNA in PCa and normal tissues. Although no significant difference of DND1 between prostate cancer tissues and normal tissues was observed, the DND1 mRNA expression level of DND1 in PCa tissues was higher than noncancerous prostate tissues based on the datasets (DND1 with higher log2FC than preset thresholds are considered differentially expressed genes, in TCGA data) as shown in Figure 1. Meanwhile, the results of immunohistochemistry also support this conclusion.

### 3.2. DND1 Protein Level in PCa Tissues

**Figure 2:**
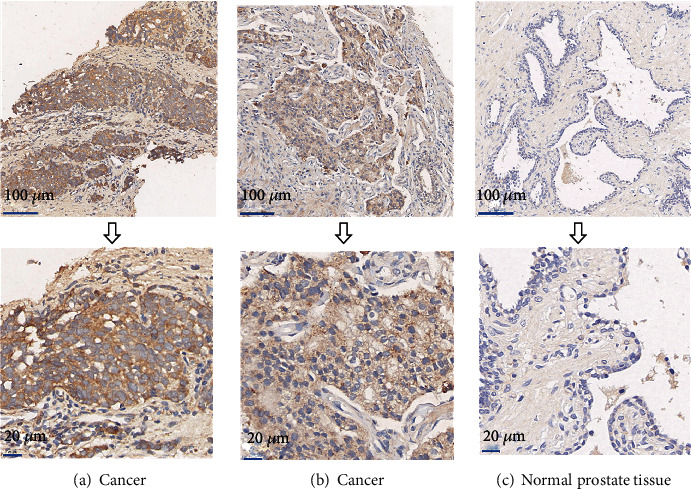
Immunostaining of DND1 in prostate cancer and adjacent normal prostate tissues. (a) Immunostaining showed strong positive DND1 in the cytoplasm of prostate cancer cells. (b) Immunostaining showed moderate positive DND1 in the cytoplasm of prostate cancer cells. (c) DND1 was negatively expressed in adjacent normal prostate tissues.

The expression of DND1 at the protein level was detected in the prostate targeted biopsy specimen from 83 PCa patients in our institution. As shown in Figure 2, there is a strong expression of DND1 in the cytoplasm in poorly differentiated prostate cancer, while there is a moderate expression of DND1 in the cytoplasm in well-differentiated prostate cancer. All specimens were examined; we found that DND1 expression was elevated in 54 out of 83 patients (65.06%) and expression was low in 29 out of 83 patients (34.94%) but was almost negatively or weakly expressed in paracancerous tissues. The expression of DND1 in tumor tissues was higher than in paracancerous tissues as shown by using statistical analysis (Table 1, *P* < 0.001).

### 3.3. Association between DND1 Expression and Clinicopathological Parameters

The relationship between DND1 expression and commonly used clinicopathological characteristics in PCa is presented in Table 2. Based on patient clinical data and immunohistochemical findings from our biopsy specimens, we found that the overexpression of DND1 was related to the higher clinical stage (*P* = 0.006), ISUP grading group (*P* < 0.001), and seminal vesicle invasion (*P* = 0.006). Nevertheless, there was no evidence indicating correlations between DND1 expression and age (*P* = 0.065), preoperative PSA (*P* = 0.271), lymph node metastasis (*P* = 0.087), or bone metastasis (0.596). As shown in Table 3, there is a positive significant correlation between DND1 expression and PSA density according to the analysis of the data of 67 patients, and the correlation coefficient (*r*_s_) is 0.379 (*P* = 0.002).

### 3.4. DND1 Was a Poor Prognostic Indicator after Radical Prostatectomy

**Figure 3:**
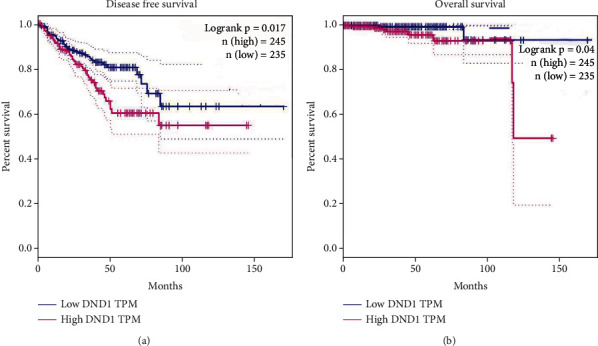
Kaplan-Meier survival analysis of (a) disease-free survival and (b) overall survival for DND1 expression in prostate cancer.

Disease-free survival and overall survival are the most vital parameters concerned for PCa patients after radical prostatectomy. To explore whether DND1 mRNA expression level is linked with disease-free survival and overall survival in prostate patients, we used the Kaplan-Meier curve method to analyze the relationship between them by using TCGA dataset. As shown in Figure 3, we found that the disease-free survival (*P* = 0.017, in TCGA dataset) and overall survival (*P* = 0.04, in TCGA dataset) were dramatically different between the DND1 high group and the DND1 low group.

## 4. Discussion

The diagnosis of prostate cancer mainly depends on PSA screening and prostate puncture biopsy, which has great financial pressure and intensive physical pain (8). Prostate cancer grows slowly and generally has a good prognosis (18). However, most patients with prostate cancer develop castration-resistant prostate cancer within 5 years after diagnosis (19). Despite available treatment options such as abiraterone acetate, enzalutamide, radiopharmaceuticals (223 radium), immunotherapy (sipuleucel-T), and chemotherapy (cabazitaxel), CRPC is still a fatal disease (20). Therefore, the emerging dilemma of the current urooncological field is to find effective prognostic biomarkers for better understanding PCa (21), thereby improving urologists' ability to diagnose and treat prostate cancer.

Our study provided evidence for the first time that DND1 could play a critical role in the progression of prostate cancer. First, we found a significant differential expression in DND1 mRNA level between PCa tissues and paracancerous tissues from TCGA dataset by bioinformatics analysis. Then, we analyzed DND1 expression at the protein level by immunohistochemistry using 83 prostate targeted biopsy specimens of PCa patients. Subsequently, we evaluated the correlation between the expression level of DND1 in the protein and clinical-pathological characteristics of PCa patients. We confirmed that high DND1 expression was significantly related to higher Gleason score, clinical stage, seminal vesicle invasion, and poor survival, but not with age, preoperative PSA level, and lymph node metastasis. Moreover, we used GEPIA to analyze the disease-free survival and overall survival of 480 PCa patients from TCGA database by the Kaplan-Meier method; the data suggested that high DND1 expression was associated with poor disease-free survival and overall survival. These findings indicated that DND1 might be a therapeutic biomarker for prostate cancerous patients and may provide a constructive treatment plan for urologists in prostate cancer. Commonly, lymph node metastasis means the higher clinical stage, and in our study, the higher clinical stage is closely related to the expression level of DND1. However, interestingly, this study did not find a significant correlation between lymph node metastasis and DND1 expression. This result may be due to the small sample size of prostate targeted biopsy and the difference in the process of lymph node dissection.

In 2005, Youngren et al. published their major historic study that Ter mutation causes primordial germ cell loss in all genetic backgrounds and testicular germ cell tumors by introducing a termination codon in the dead-end gene (22). In a follow-up study, Kedde et al. found that the function of miR-430 and miR-372 was suppressed because of the combination of DND1 and URRs located within the mRNAs (23). It provided evidence that DND1 maintains germ cell viability and inhibits the formation of germ cell tumors by counteracting microRNA-mediated silencing of mRNAs. Recent studies have shown that microRNA-24-mediated suppression of DND1 suppressed the proliferation of gastric cancer cells and CRC cells (16). Similarly, the miR-24-mediated downregulation in DND1 expression level inhibited the expression of cyclin-dependent kinase inhibitor 1B (CDKN1B) and resulted in enhanced proliferation and reduced apoptosis in tongue squamous cell carcinoma (TSCC) cells (24). And the expression of DND1 was significantly upregulated in gastric cancer cells, CRC cells, and TSCC cells. These findings are consistent with our results acquired by immunohistochemistry. However, contrary to these studies, some findings showed that the expression of DND1 in some tissues will inhibit cell proliferation and metastasis and prevent the occurrence of cancer. For example, research from Cheng et al. has established that Dnd1 regulates the expression of Bim to facilitate apoptosis via the competitive combination with miR-221 in breast cancer (25). Another case in this point goes to Wampfler et al. who have shown that the expression of the RNA-binding proteins RBM38 and DND1 is suppressed in primary AML patients (15). Consistent with the above studies, DND1 counteracts the competitive inhibitory effect of miRNA on mRNA by binding to LATS2 and increasing the expression level of LATS2 in hepatocellular carcinoma to play a tumor suppressor role (17). Overall, these studies indicate that the occurrence of cancer is related to the function and content of microRNA and DND1-mediated mRNA, and the content of mRNA is regulated by the competitive inhibition of microRNA and DND1. Moreover, excitingly, Yamaji et al. showed that DND1 destabilizes target mRNAs via direct recruitment of the CCR4-NOT deadenylase (CCR4) complex to regulate mRNA content in a different way (26). Despite these promising results, there are still many unanswered questions about the regulation of DND1.

To date, Lynch et al. provide evidence that miR-24 inhibits the occurrence of prostate cancer by targeting p27 (CDKN1B) and p16 (CDK2NA) in some way (27). Kedde found that Dnd1 regulates microRNA access to target mRNA. And our study suggested that DND1 is overexpressed in prostate cancer. Further research should be carried out to establish the relationship between miR-24, DND1, and P27 in prostate cancer. Meanwhile, our study data concluded that high DND1 expression was significantly associated with advanced clinicopathological features and poor prognosis in PCa patients.

## 5. Conclusions

We demonstrated that DND1 was overexpressed in patients with PCa. We further confirmed that high DND1 expression levels were associated with relatively higher ISUP grading groups, clinical stage, and seminal vesicle infiltration. Finally, we found that upregulation of DND1 predicted shorter disease-free survival and overall survival in patients who had undergone radical prostatectomy in TCGA dataset. Therefore, DND1 can be used as a good biomarker in the diagnosis and prognosis of patients with prostate cancer. However, due to the small sample size derived from prostate targeted biopsy, it is necessary to expand the sample size and draw material from the resected prostate in radical prostatectomy to further verify the authenticity of our research results. Moreover, the underlying mechanism of an interesting phenomenon that there was strong DND1 expression in the luminal epithelium of most well-differentiated prostate cancer but there is almost no expression of DND1 in some poorly differentiated prostate cancer is still unclear. Therefore, to further figure out the role of DND1 as a novel prognostic factor in PCa patients, more in-depth research and follow-up are ought to be launched to verify the reliability of our research results.

## Figures and Tables

**Table 1:** Analysis of DND1 expression level between prostate cancer tissues and paracancerous tissues.

Test statistics^a^	
	Paracancerous tissues-PCa
*Z*	-7.871^b^
Asymptotically significant (two-tailed)	*P* < 0.001^∗^

^a^Wilcoxon signed-rank test. ^b^Based on positive rank. ^∗^ indicates *P* < 0.05 with statistical significance.

**Table 2:** Clinicopathologic variables and DND1 expression in 83 prostate cancer patients.

Variable	Group	DND1 expression			*P* value^#^
		*n*	Low	High	
Age	<70	55	23 (27.7%)	32 (38.6%)	0.065
	≥70	28	6 (7.2%)	22 (26.5%)	

Perioperative PSA	≤10	20	9 (10.8%)	11 (13.3%)	0.279
	>10	63	20 (24.1%)	43 (51.8%)	

Clinical stage	T2	22	13 (15.7%)	9 (10.8%)	0.006^∗^
	T3-4	61	16 (19.3%)	45 (54.2%)	

ISUP grading group	ISUP1-2	41	27 (32.5%)	14 (16.9%)	<0.001^∗^
	ISUP3-5	42	2 (2.4%)	40 (48.2%)	

Seminal vesicle invasion	Absence	12	0 (0%)	12 (14.5%)	0.006^∗^
	Presence	71	29 (34.9%)	42 (50.6%)	

Lymph node metastasis	Absence	6	0 (0%)	6 (7.2%)	0.087
	Presence	77	29 (34.9%)	48 (57.8%)	

Bone metastasis	Absence	6	1 (1.2%)	5 (6%)	0.596
	Presence	77	28 (33.7%)	49 (59.0%)	

^#^ *P* value was analyzed by the chi-square test. ^∗^*P* < 0.05 with statistical significance.

**Table 3:** Correlation between DND1 expression and PSA density in 67 prostate cancer patients.

Correlation				
			DND1 expression level	PSA density
Spearman rho	DND1 expression level	Correlation coefficient	1.000	0.379^∗∗^
		Significance (two-tailed)	—	0.002
		Number of cases	67	67
	PSA density correlation coefficient	Correlation coefficient	0.379^∗∗^	1.000
		Significance (two-tailed)	0.002	—
		Number of cases	67	67

^∗∗^correlation is significant at the 0.01 level (two-tailed).

## Data Availability

The data used to support the findings of this study are available from the corresponding author upon request.
